# Organic Electro-Optic Materials with High Electro-Optic Coefficients and Strong Stability

**DOI:** 10.3390/molecules29133188

**Published:** 2024-07-04

**Authors:** Shuhui Feng, Shuangke Wu, Weijun Zhang, Fenggang Liu, Jiahai Wang

**Affiliations:** School of Chemistry and Chemical Engineering, Guangzhou University, Guangzhou 510006, China; 32105100008@e.gzhu.edu.cn (S.F.); 1905100045@e.gzhu.edu.cn (S.W.); 32105100028@e.gzhu.edu.cn (W.Z.)

**Keywords:** nonlinear optical material, chromophore, electro-optic coefficient

## Abstract

The preparation of high-performance electro-optical materials is one of the key factors determining the application of optoelectronic communication technology such as 5G communication, radar detection, terahertz, and electro-optic modulators. Organic electro-optic materials have the advantage of a high electro-optic coefficient (~1000 pm/V) and could allow the utilization of photonic devices for the chip-scale integration of electronics and photonics, as compared to inorganic electro-optic materials. However, the application of organic nonlinear optical materials to commercial electro-optic modulators and other fields is also facing technical bottlenecks. Obtaining an organic electro-optic chromophore with a large electro-optic coefficient (r_33_ value), thermal stability, and long-term stability is still a difficulty in the industry. This brief review summarizes recent great progress and the strategies to obtain high-performance OEO materials with a high electro-optic coefficient and/or strong long-term stability. The configuration of D-π-A structure, the types of materials, and the effects of molecular engineering on the electro-optical coefficient and glass transition temperature of chromophores were summarized in detail. The difficulties and future development trends in the practical application of organic electro-optic materials was also discussed.

## 1. Introduction

In the past decade, driven by emerging technologies such as 5G wireless communication, big data, artificial intelligence/machine learning (AI/ML), cloud services, telemedicine, autonomous vehicles, and the demand for telework caused by the COVID-19 epidemic, the world has experienced an explosive growth in Internet data traffic. Information processes and transmission materials using photons as carriers have shown broad application prospects in fields such as 5G communication, radar detection, terahertz, and electro-optic modulators. One of the key factors determining the application of optoelectronic communication technology is the preparation of high-performance electro-optical materials.

At present, the electro-optic materials used commercially are mainly inorganic materials represented by lithium niobate [1]. In particular, the film lithium niobate technology that has emerged in recent years is to make lithium niobate crystals into film, and the film lithium niobate is bonded to the silicon layer. The electro-optical coefficient of the film lithium niobate is almost the same as that of the bulk lithium niobate. Compared with the bulk lithium niobate, the film lithium niobate provides the advantage of flexible waveguide design. The refractive index difference between the core layer and the cladding layer is large, which is more conducive to restricting the light field, making the light spot of the waveguide smaller, and is conducive to increasing the intensity of the modulation electric field. Therefore, thin film lithium niobate technology can produce smaller Mach-Zehnder modulation MZM, and the modulation rate can reach 70 GHz. Based on lithium niobate MZM, by doping, the refractive index is changed to restrict light and form an optical waveguide. However, due to the small doping concentration, small change in refractive index and large light spot of the optical waveguide, the photoelectric coefficient of lithium niobate was only 30 pm/V, and the bulk lithium niobate photoelectric modulator is huge, requiring traveling wave modulation, complex design, and high power consumption. The lithium niobate material itself has a series of insurmountable shortcomings, such as a low electro-optic coefficient, difficult crystal growth and processing, etc. [2].

**Figure 1:**
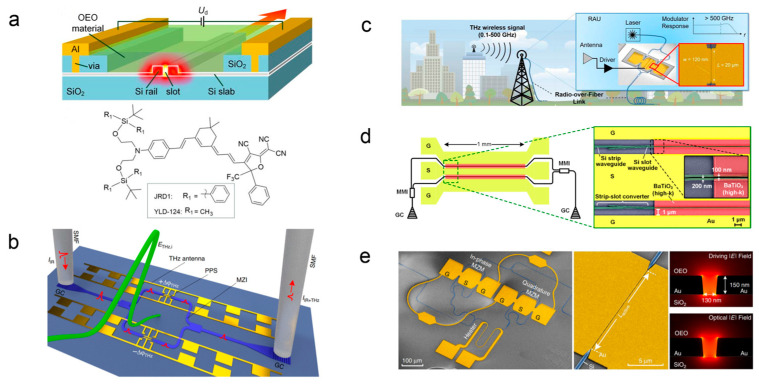
(**a**) SOH MZM modulator based on organic electro-optic materials [30], (**b**) terahertz field detector [14], (**c**) sub-THz microwave [8], (**d**) SOH silicon MZM modulator [31], and (**e**) IQ modulator [32].

After years of development, the advantages of organic electro-optic materials have become increasingly apparent. Compared with inorganic materials, organic electro-optic materials have advantages such as a high electro-optic coefficient, high optical damage threshold, fast response speed, high bandwidth, low dielectric constant, good processability and integration, low cost, and wide selection range [3]. Moreover, they can easily integrate with semiconductor microelectronic devices, thus having great application prospects [4]. As shown in Figure 1, many journals such as Science and Nature [5,6,7] reported that research groups such as Juerg Leuthold of the Zurich University of Technology in Switzerland and other groups have used a CLD-type organic second-order nonlinear optical chromophore to prepare a 500 GHz electro-optic modulator [8], terahertz field detector, and other optoelectronic devices [9,10,11,12,13,14,15,16]. The United States and Europe have also set up companies to produce organic electro-optic chromophore and related optoelectronic devices. All of these indicate that the organic second-order nonlinear chromophore has a broad practical prospect [17,18,19].

However, the application of organic nonlinear optical materials to commercial electro-optic modulators and other fields is also facing technical bottlenecks; it is difficult to meet the Telecordia GR-468-CORE standards [20]. Obtaining an organic electro-optic chromophore with a large electro-optic coefficient (r_33_ value), thermal stability, and poling orientation stability is still a difficulty in the industry [21].

**Figure 2:**
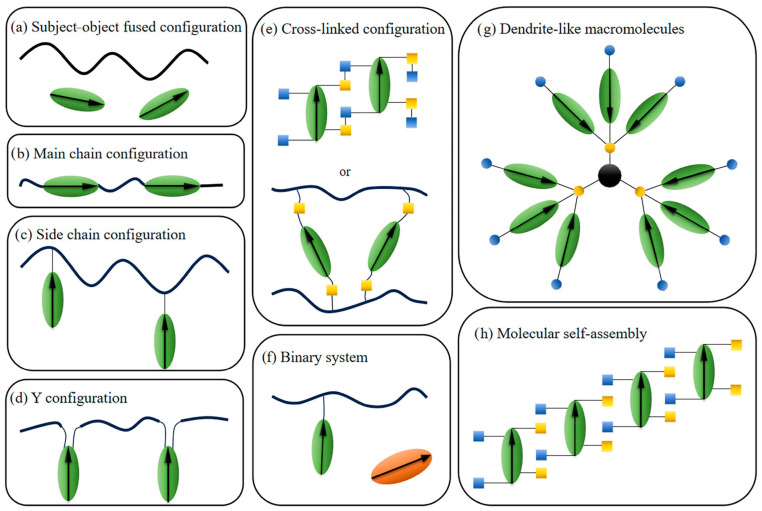
Common types of organic electro-optic materials.

With the development of materials science, nonlinear optics, communication science, and other fields, organic nonlinear optical materials have gradually attracted the attention of scientists. In the early research, the relationship between chromophore structure and performance was still not clear, the electro-optic coefficient was low, and the problems of optical loss and stability have not been solved. As research deepens, as shown in Figure 2, various types of organic electro-optical materials are gradually developed. Especially since 2000, the electro-optic coefficient of organic electro-optic materials has significantly increased, and the thermal stability has also been enhanced. Representative work in this area comes from the L R. Dalton research group of Washington University [22,23], the Alex Jen and Jingdong Luo group of City University of Hong Kong [24,25], the Li Zhen Research Group of Wuhan University [26,27,28], and the Yokoyama Research Group of Kyushu University [29], etc.

## 2. Categories of Organic Electro-Optical Materials

As shown in Figure 2, so far, the common electro-optic polymer systems were as follows: host–guest doped type, main chain type, side chain type, cross-linked type, hyperbranched dendrimer, self-assembled molecular glass, etc. These systems each have their own unique characteristics, as well as their own advantages and disadvantages.

(a) Host–guest doping type

Generally, a 25 wt% of chromophore molecules was physically doped into a 75 wt% of commercial polymethyl methacrylate (PMMA) or polycarbonate (APC) [33]. This kind of material has the advantage of simple preparation. However, due to the lower chromophore doping concentration, the electro-optical coefficient was usually not high. [34] Meanwhile, due to the relatively low glass transition temperature and weak poling orientation stability, it is difficult to meet the operational requirements of devices at high temperatures [35,36].

(b) Polymer type

The polymer materials mainly divided into main chain polymers and side chain polymers [37,38,39]. The stability of polymer systems was relatively high, with a higher glass transition temperature [40]. However, polymeric materials usually require a large amount of chromophore materials, and to some extent, the demand for chromophores was very high during the synthesis process [41]. Although it is relatively complex to synthesize, there have been reports of highly stable side chain polymers with large r_33_ values used in optoelectronic devices which have been running stably for 2000 h and still maintain an electro-optic coefficient of over 90% of the original value [29].

(c) Hyperbranched Dendritic Molecules

Due to the hyperbranched structure, the electrostatic interaction between molecules was greatly weakened so as to achieve a large electro-optic coefficient [42]. The electro-optic coefficients of the hyperbranched materials were much higher than that of ordinary chromophores [43,44,45]. However, hyperbranched structures also have their drawbacks: due to the lack of polymer introduction, the thermal stability of the material is relatively poor. The glass transition temperature of these molecules was usually lower than 120 °C [43,46,47].

(d) Self-assembling type

The self-assembled molecular glass uses intermolecular forces such as hydrogen bonds or π–π stacking to solidify oriented chromophores during poling, which greatly improves the stability of the electro-optic coefficient. However, the disadvantage is that the synthesis was relatively complex, since functional groups that can generate intermolecular force need to be introduced into the chromophore. This type of material usually has a large electro-optic coefficient and long-term alignment stability at room temperature. However, the self-assembled materials showed insufficient long-term alignment stability at high temperatures above 85 °C [48,49].

(e) Cross-linked type

**Figure 3:**
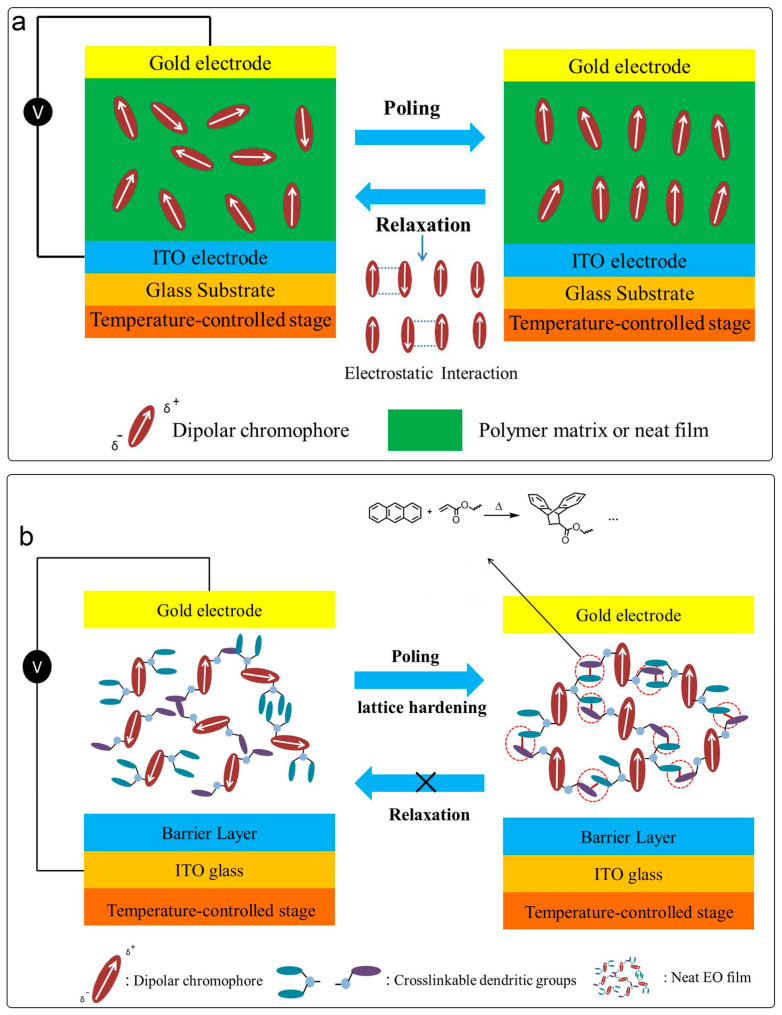
(**a**) Poling process of traditional chromophore. (**b**) Poling and curing process of cross-linked chromophore.

The cross-linked materials gradually form a network of polymer structures during poling and heating processes. As shown in Figure 3, after poling is completed, increases in the temperature and the Diels−Alder cross-linking reaction occur, forming a polymer network that immobilizes the polarized oriented molecules and greatly improves the long-term alignment stability [50,51]. However, the design of cross-linking systems is relatively complicated, the matching between cross-linking temperature and chromophore must be taken into account, and the poling process is also very complicated [52,53,54].

Although various high-performance electro-optic materials have been designed, the practical application of organic electro-optic materials still faces many challenges. Understanding how to obtain an organic electro-optic chromophore with a large electro-optic coefficient (r_33_ value), thermal stability, and polarization orientation stability is still the focus of research. In order to apply organic electro-optic materials to high-performance commercial devices, the chromophore must have a large electro-optic coefficient and a high glass transition temperature [55].

Chromophore molecules need to be polarized under the effect of an electric field, showing a non-centrosymmetric arrangement, in order to show electro-optic activity. The electrostatic interactions between molecules can hinder this ordered arrangement, thereby reducing the efficiency of converting hyperpolarizability values into macroscopic electro-optic coefficients [56,57]. The electro-optic coefficient of the chromophore was usually improved by large first-order hyperpolarizability, the introduction of a steric hindrance group, and the design of dendritic molecular configuration [58].

The long-term stability of the electro-optic coefficient was limited by the glass transition temperature of the chromophore. At high temperatures, especially when the temperature exceeds the glass transition temperature of the chromophore, the molecules of the chromophore that have been orderly arranged under the action of the electric field will decline to the disordered isotropic arrangement due to the electrostatic interaction, and the electro-optic coefficient will also decline. Therefore, the preferable glass transition temperature of chromophore materials is higher than 200 °C to meet the requirements for commercialized devices.

## 3. Chromophores with High Electro-Optic Coefficients

The second-order nonlinear chromophore consists of three parts: electron donor (D), electron bridge (π) and electron acceptor (A), which was commonly known as the D-π-A structure. These three components could have a significant impact on the performance of molecules. With the deepening of research, the structure of chromophores has been continuously optimized. The donor of second-order nonlinear chromophores was composed of electron-rich compounds. They often contain heteroatoms such as nitrogen, oxygen, and sulfur. The electrons were transferred by donor through the movement of n-π * charges, enhancing the mobility of π electrons within the molecule. When selecting electron donors, factors such as electron-donating ability and thermal stability should be considered.

**Figure 4:**
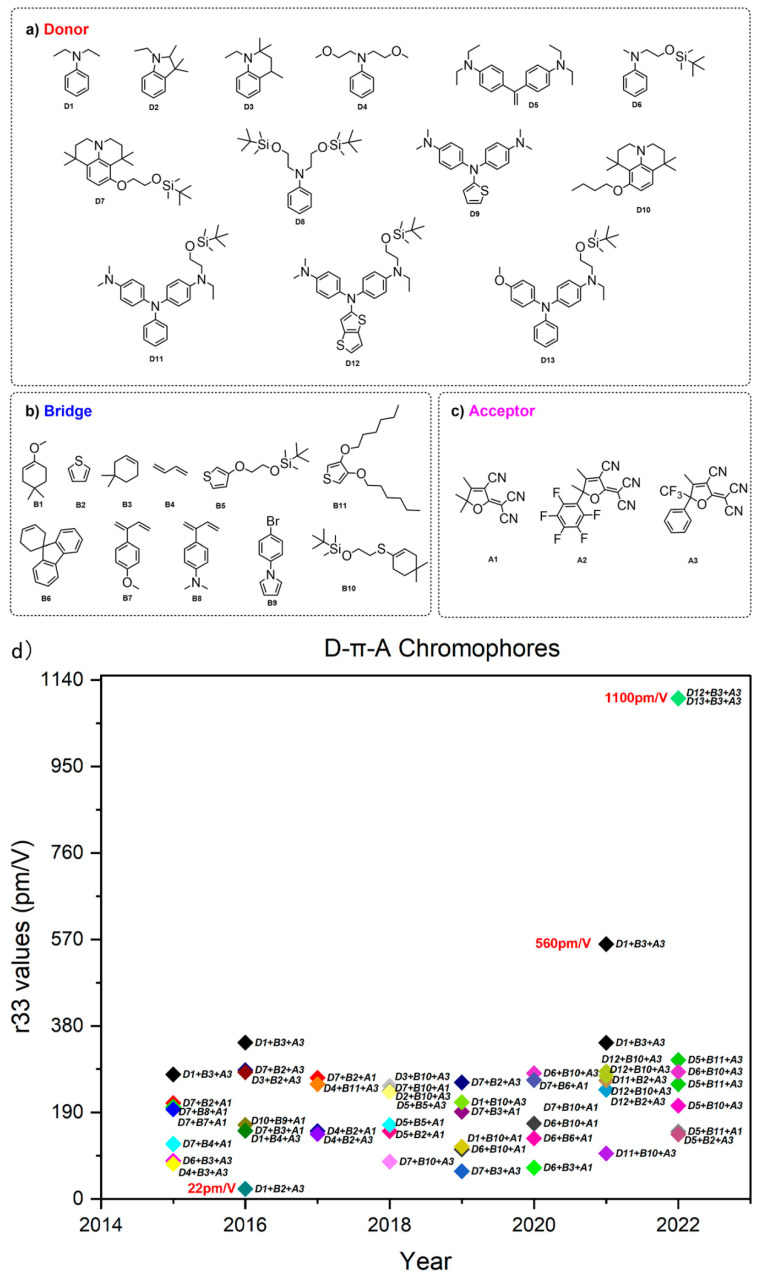
(**a**–**c**). Combination of donor, acceptor, and bridge of chromophores with large electro-optic coefficients. (**d**) The electro-optic coefficient of chromophores varies with years.

After extensive experiments, the aniline derivatives were the most suitable donor groups considering factors such as the electron-donating ability of the donor, the ease of synthesis, and stability. Aniline derivatives have strong electron-donating ability and easy synthesis [59]. As shown in Figure 4, however, there were also significant differences in their performance. Compared to ordinary aniline D1 [59], trimethylindoline D2, tetrahydroquinoline D3 [60], and Jiuluonidine derivative [61,62] D7 exhibit stronger electron-donating ability, resulting in larger hyperpolarizability and electro-optical coefficients. The usual method to improve the electron-donating ability of donors was to introduce electron-rich groups, which were usually introduced in the ortho and para positions of the benzene ring. In order to further enhance the electron-donating ability of the donor, a research group also designed the double donors structure D5 [63], which uses two ordinary aniline donors as donor groups for the chromophore [63,64,65,66]. This special design not only enhances the electron-donating ability of the donor, but also acts as a steric hindrance group, thus further improving the electro-optic coefficient.

The chromophore with triphenylamine as the donor has a smaller first-order hyperpolarizability compared to the chromophore with an alkyl amine as the donor. This is because the electrons on the nitrogen atom will delocate to two other benzene rings that are not in the same plane as the chromophore, thereby weakening the electron-giving ability. However, due to their rigid structure, chromophores with triphenylamine as a donor are often more stable than alkyl amines. In order to overcome the lower first-order hyperpolarizability, atomic-rich groups such as oxygen are often introduced into triphenylamine to enhance its electron-donating ability, such as in D10 and D12. In addition, replacing the benzene ring in D9 and D11 with heterocyclic compounds such as thiophene can also enhance the electron-donating ability [67].

The conjugated electron bridge of the chromophore plays a role in connecting electron donors and electron acceptors. The electron bridge plays a very important role on the µβ value of chromophores; as the length of the conjugated bridge increases, the value of the chromophore’s β will also increase. Of course, if the conjugation length reaches a certain value, the first-order hyperpolarizability will also display saturation phenomenon. At the same time, the aromaticity of the electronic bridge can also affect the β value of chromophores. Due to its conjugated structure, the benzene ring has become one of the earliest conjugated bridges used, but due to its strong aromaticity and large electron delocalization energy, its electron transfer ability is relatively weak. For pentacyclic furan, thiazole, and other compounds, the introduction of heteroatoms such as N, O, and S not only reduces their aromaticity, but also increases their electron density relative to the benzene ring due to the introduction of atomic-rich groups, resulting in a decrease in delocalization energy and an increase in the first-order hyperpolarizability of the molecule [68]. Among the five-membered heterocycles, thiophene is the most commonly used chromophore, [69,70] mainly due to its stability due to the furan and pyrrole groups [61]. Non-aromatic polyene structures have the strongest ability to transport electrons. According to the bond length change theory, non-aromatic carbon double bonds can obtain lower BLA values, leading to a higher first-order hyperpolarizability of molecules. However, due to the presence of cis-trans isomerism, the optical and chemical stability of double bonds is often poor [71]. The use of circular configurations to fix double bonds in trans configurations is a commonly used solution. It achieved 100% trans structure by introducing long-chain alkyl groups with steric hindrance groups on isophorone (CLD) [72]. At present, the modification of electronic bridges mainly focuses on the modification of thiophene bridges and CLD bridges [73]. Of course, in addition to the inherent characteristics of the electron bridge, the position of the electron bridge, the electron donor, the electron acceptor, and the reasonable optimization combination between the electron bridges can achieve greater first-order hyperpolarizability and electro-optic coefficient.

Electron acceptors were generally composed of functional groups with strong electron-withdrawing ability and possess strong electron-withdrawing ability [74]. The earliest electronic receptors used include simple receptors such as nitro (–NO_2_), aldehyde (–CHO), and cyano (–CN). In subsequent studies, some novel receptors with strong electron-withdrawing abilities emerged, such as SDS, TCI, BBA, and TCV [74,75]. However, the electron-absorption ability of these acceptors still cannot meet the needs of larger first-order hyperpolarizability of chromophores. In the late 1990s, a new type of electron acceptor, trinitrofuran (TCF) [76] emerged. TCF is formed by connecting three electron-withdrawing groups, CN groups on furan, and the molecule has a planar conjugated structure, thus possessing strong electron-withdrawing ability. Since its synthesis, this molecule has been widely used in the structure of chromophores and has achieved excellent performance. Due to its good processability and stability, as well as its strong electron absorption ability, researchers have prepared organic electro-optic modulators with good performance, such as a high bandwidth and low half wave voltage, using some chromophores using TCF as acceptors [77,78].

By continuously adjusting the substituent groups of TCF receptors, people have synthesized electron acceptors with better performance, and their electron-absorption ability has also been continuously enhanced. Replacing the methyl group of the TCF side chain with a trifluoromethyl group (-CF_3_) has formed a new type of CF_3_-TCF receptor system [79]. This type of acceptor has a significantly improved electron-absorption ability and solubility compared to TCF receptors. Many chromophores using CF_3_-TCF as acceptors can even form films independently without the use of polymers [80]. In addition, optimizing the R2 group in CF_3_-TCF to include a benzene ring, thiophene, and other groups can further enhance the isolation effect, weaken the electrostatic interaction between molecules, and thereby improve the macroscopic electro-optic coefficient [79,81].

We summarized the effects of the combination of donors, acceptor, and bridges on the electro-optic coefficients of chromophores, especially for chromophores with an electro-optic coefficient greater than 200 pm/V. From Figure 4, we can obtain some information: within a certain range, the electro-optical coefficient of chromophores increases with the intensity of the donor and acceptor. CLD bridges have better electron transfer capabilities than thiophene bridges, resulting in larger electro-optical coefficients. In the past decade, the electro-optic coefficients of most chromophores have not increased significantly, and the electro-optic coefficients of most large chromophores were less than 300pm/V. Of course, what is surprising is that with the emergence of new triphenylamine derivative donors, this situation has changed. Triphenylamine itself is a weak donor, but its stability is strong. Introducing electron-rich atoms such as N or O into triphenylamine derivatives to form donors D11 and D12 can effectively increase the electron-donating ability of the donor. Combined with the introduction of the BCB layer, the electro-optical coefficient of the chromophores with D11 and D12 donors exceeds 1000 pm/V [23].

**Figure 5:**
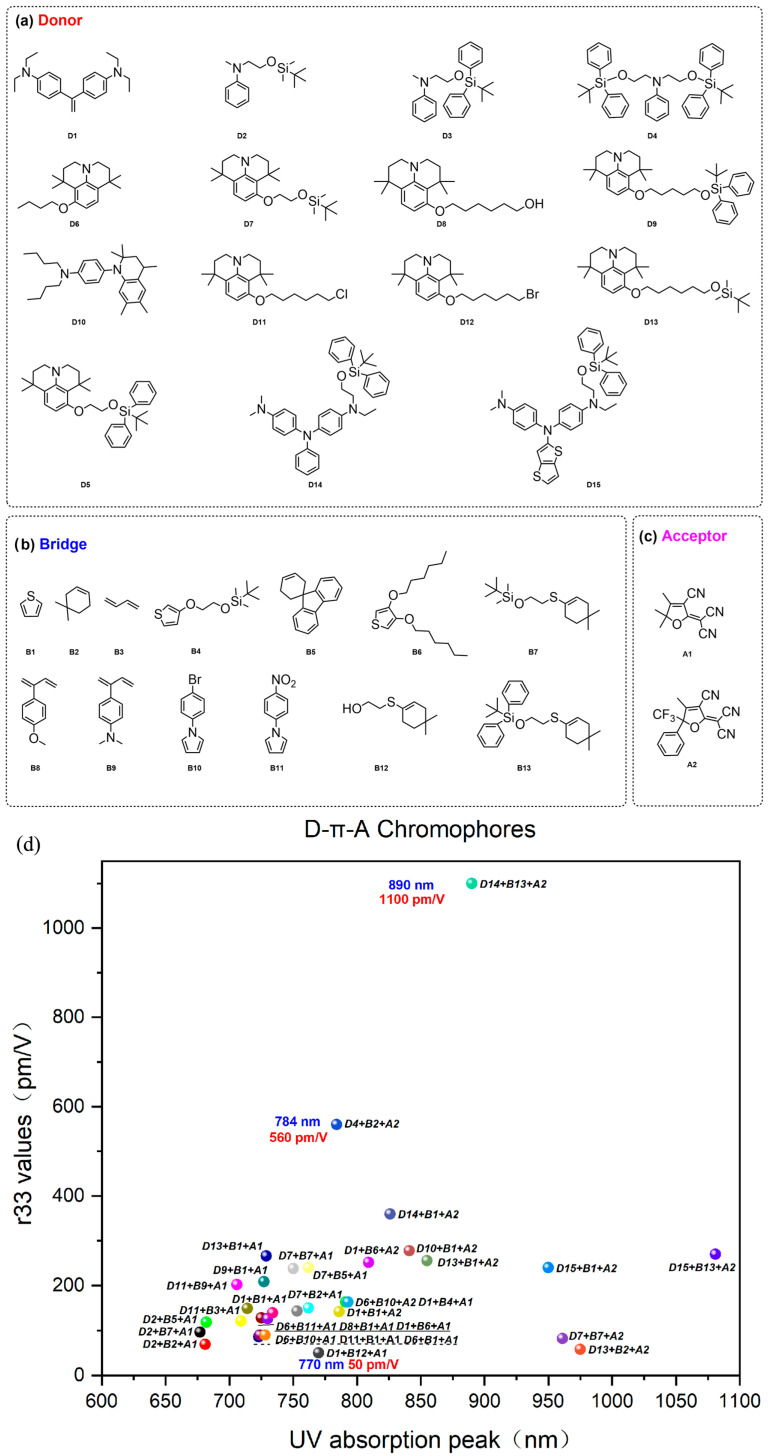
(**a**–**c**) Combination of donor, acceptor, and bridge of chromophores with large electro-optic coefficients. (**d**) The electro-optic coefficient of chromophores varies with maximum UV absorption wavelength.

We also summarized the relationship between the electro-optical coefficient of chromophores and the maximum absorption wavelength of ultraviolet radiation. From Figure 5, it can be seen that the electro-optical coefficient of the chromophore is not positively correlated with the maximum ultraviolet absorption wavelength. The optimal UV absorption wavelength is between 780 and 890 nm. An absorption wavelength that is too short can cause a low first-order hyperpolarizability of the chromophore, while an ultraviolet absorption that is too red-shifted may cause an absorption of the chromophore at 1310 nm, resulting in optical loss.

**Figure 6:**
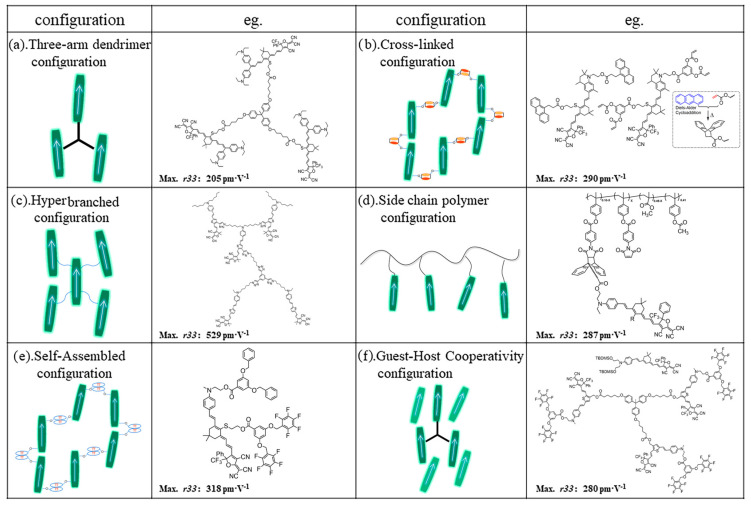
(**a**) Three-arm dendrimer; (**b**) cross-linked chromophore configuration; (**c**) hyperbranched configuration; (**d**) side chain polymer configuration; (**e**) self-assembled configuration; and (**f**) guest–host cooperativity configuration.

To obtain a large electro-optic coefficient, there are several other methods besides designing and synthesizing chromophores with high first-order hyperpolarizability. Examples shown in Figure 6 include binary guest–host systems [82], dendritic structures, hyperbranched structures [43,83,84], self-assembly systems [85,86], side chain polymer systems, binary cross-linked chromophores [87], and other methods. These branched structures can effectively weaken the electrostatic interactions between molecules, improving the poling efficiency to obtain large electro-optical coefficients [88,89]. In addition, intermolecular hydrogen bonding can also help improve poling efficiency. Of course, despite their large electro-optical coefficients, the difficulty in synthesizing these branched chromophores is also a drawback. Moreover, the glass transition temperature of these branched chromophores is usually not high (below 120 °C), and it is necessary to increase the glass transition temperature with the help of cross-linking or rigid groups.

## 4. Chromophores with High Glass Transition Temperature

**Figure 7:**
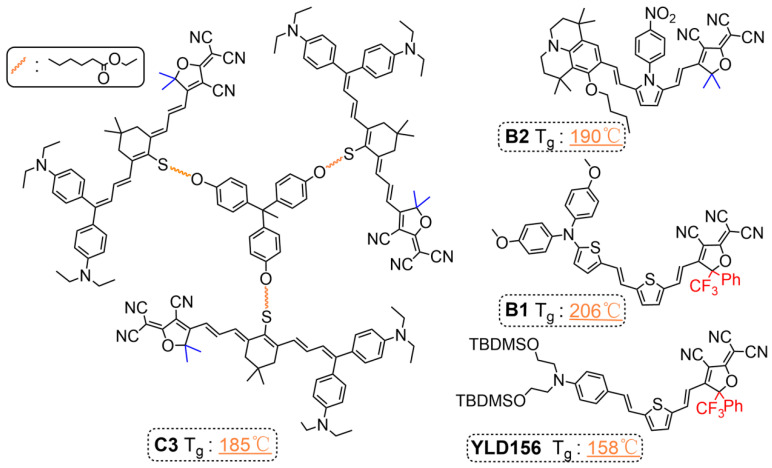
Single chromophores with a glass transition temperature exceeding 150 °C.

For a single chromophore, there are very few chromophores with a glass transition temperature exceeding 150 °C as shown in Figure 7 [61,90]. The chromophore with TCF as the acceptor usually has a higher glass transition temperature than chromophore with a CF_3_-TCF acceptor. Some literature has reported that these chromophores have high glass transition temperatures, but this is essentially meaningless because chromophores with TCF as acceptors usually cannot form films independently and require the help of polymers such as PMMA and APC [33].

For chromophores with CF_3_-TCF as an acceptor, the glass transition temperature was usually between 60 and 120 °C, which leads to a poor long-term poling orientation stability of pure chromophores [81]. Some CF_3_-TCF-based chromophores with rigid groups have high glass transition temperatures, such as the T_g_ of YLD156, reaching 158 °C, while the glass transition temperature of chromophore B1 is higher than 200 °C [67]. However, excessive glass transition temperature is also detrimental to poling, as high temperatures may cause the decomposition of some chromophores.

Except for single chromophores, there are currently two types of materials that may meet the long-term stable operation of commercial devices, especially at high temperatures: cross-linked materials and side chain polymer materials. However, there are not many reports on cross-linked electro-optic materials, and many are in exploratory research. At present, there are three types of cross-linking agents for cross-linked materials: polymer cross-linking agents, small molecule cross-linking agents, and chromophore cross-linking agents. Early research mainly focused on polymer cross-linked materials.

**Figure 8:**
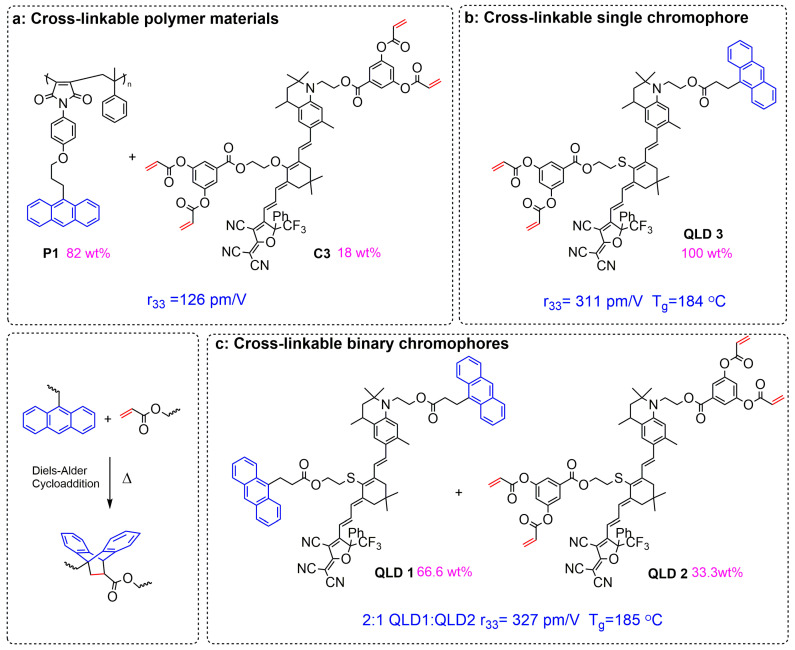
Comparison of structural properties of various cross-linked materials ((**a**) traditional polymer cross-linked materials; (**b**) unit cross-linked chromophores; and (**c**) binary cross-linked chromophores) [52,53,54].

As shown in Figure 8, usually, cross-linkable groups including thermal cross-linking and photo cross-linking agents were introduced into the polymer and chromophore, respectively [54,91,92]. In this case, the mass fraction of chromophore was usually less than 25wt%, and the electro-optic coefficient of the cross-linked system is subject to the content of chromophore, which was usually less than 150 pm/V [52,93]. However, due to the introduction of polymer, the glass transition temperature after cross-linking can be higher than 200 °C [50]. The other kind of cross-linking agent was the small molecule cross-linking agent, whose molecular weight was much smaller than that of the polymer cross-linking agent, which can effectively increase the content of chromophore, thus obtaining a greater electro-optic coefficient than that of the polymer type. However, the electro-optic coefficient was still limited by the lower content of chromophore and the compatibility of small molecule cross-linkers. Due to the presence of small molecules, the glass transition temperature of such cross-linked materials was usually not high [51,94].

In order to solve this problem of a lower electro-optic coefficient, binary cross-linking materials were proposed, and cross-linkable groups were introduced into two different chromophores. The chromophores modified with anthracene and acrylate can undergo a Diels–Alder cross-linking reaction to fix the oriented chromophores through chemical bonds after poling orientation. Cross-linkable binary chromophores HLD1/HLD2 [87] and QLD1 and QLD2 [95] were developed to achieve very high maximum r_33_ values of around 300 pm/V due to the high chromophore density in the binary cross-linking system. The glass transition temperature of this type of EO film could be as high as 185 °C, which is higher than for any other pure chromophore films. The 100wt% chromophore content and the effective occurrence of a cross-linking reaction enable the system to achieve a balance between the electro-optical coefficient (∼300 pm V^−1^) and the glass transition temperature (∼180 °C).

Binary cross-linked materials have been applied to many advanced optoelectronic devices and have great potential for commercial applications. However, the design of two-component cross-linking materials involves the matching of the glass transition temperature and ratio of two chromophores. If the glass transition temperature of two chromophores varies greatly, one of the chromophores is polarized while the other chromophore is not oriented, which will reduce the poling efficiency. Therefore, the most suitable glass transition temperature for binary cross-linked chromophores is slightly lower than the cross-linking temperature, and the glass transition temperatures of the two chromophores are similar; this is beneficial for the improvement of the electro-optic coefficient.

To avoid the problem of the complex design of binary cross-linking systems, a single-component cross-linkable electro-optic material QLD3 was proposed for the first time. The single-component cross-linked material has a similar r_33_ value (306 pm/V) and glass transition temperature (184 °C) to the binary cross-linkable material without avoiding the problem of the matching and proportioning of two-component cross-linked materials. It is worth further study to optimize the performance of single-component cross-linkable electro-optic chromophores. The fourth type is multi-chromophore cross-linked material represented by QLD4. Benefiting from the weakening of intermolecular electrostatic interactions, the electro-optical coefficient of the chromophore was further improved, and with the increase in molecular weight, the glass transition temperature is also expected to further increase. However, the complexity of the synthesis steps of this material was a major disadvantage.

There is currently limited research on neat chromophore (100 wt% chromophore) cross-linking systems. The highly efficient binary cross-linkable dendritic chromophores FZL1–FZL2 were developed by an Anthracene-maleimide Diels–Alder (DA) reaction with three cross-linking groups modified on the donor and electron bridge of the chromophores. A covalently cross-linked network was formed by a DA reaction at 135 °C after electric field poling orientation, which greatly improving the long-term alignment stability of the materials. Faster cross-linking at lower temperatures (compared to 160 °C for HLD1-2 and QLD1-2) is beneficial for energy conservation, while also preventing chromophores from being damaged at high temperatures. An electro-optic coefficient up to 266 pm/V and glass transition temperature as high as 178 °C was achieved in the cross-linked film FZL1/FZL2. [96]. Long-term alignment stability tests showed that after heating at 85 °C for over 500 h, 99.73% of the initial r_33_ value was maintained for the poled cross-linked electro-optic film 1:1 FZL1/FZL2. The excellent electro-optic coefficient and stability of the material indicate the practical application prospects of organic electro-optic materials.

**Figure 9:**
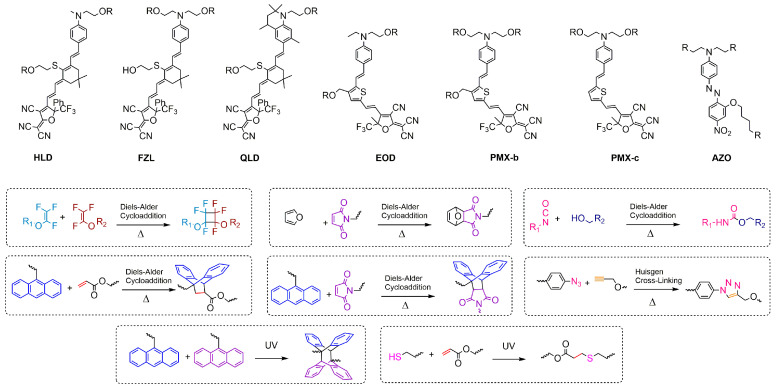
Summary of chromophore bodies and cross-linking types of cross-linked electro-optical materials.

Currently, there are very few reports on binary cross-linked chromophores (100 wt% chromophore), and the types of cross-linking reactions were limited to the Anthracene-acrylate-based Diels–Alder (DA) reaction (AA-DA) or Anthracene-maleimide-based Diels–Alder (DA) reaction (AM-DA). There were various cross-linking reactions between chromophores and polymers/small molecules, which may be suitable for binary pure chromophore cross-linking systems. The following is a summary, as shown in Figure 9, of some reaction types and chromophore main structures that can be applied to cross-linked materials: the Maleimide-furan-based Diels–Alder (DA) reaction (MF-DA), the Azide-alkyne-based Huisgen cycloaddition reaction (AA-HC) [97], trifluorovinyl ethers (TFVE) cyclodimerization [98], the thiol–ene click reaction [92], [4+4] cycloaddition between anthracene groups, the photo-cross-linking of the anthracene group, and isocyanate–hydroxyl reactions. The occurrence of the cross-linking reaction is usually a reversible process, and after a certain temperature is exceeded, the reverse cross-linking reaction will occur. Moreover, the occurrence of the cross-linking reaction is slower at low temperatures, and occurs quickly after exceeding a certain temperature. So the initial temperature (Ts) at which the cross-linking reaction occurs quickly is very important for cross-linked electro-optical materials. This temperature needs to be higher than the glass transition temperature of the chromophore, otherwise during the polarization process, the molecules will have cross-linked before they can undergo polarization turning. The cross-linking temperature varies for different reactions, and a few examples are as follows: the AA-DA reaction (120–160 °C) or AM-DA reaction (100–135 °C), MF-DA reaction (100–135 °C), AA-HC reaction (100–135 °C), and TFVE reaction (>160 °C). The best state for cross-linking initiation temperature is 20 °C–50 °C higher than the glass transition temperature of the chromophore. If the difference is too small, the molecules have already cross-linked before polarizing and turning. If the difference is too large, the molecules move too fiercely due to the high temperature. All of these will cause a decrease in poling efficiency. For the photo-cross-linking reaction, there is no temperature issue, but the influence of light on the chromophore itself must also be considered.

Different cross-linking reactions and cross-linking groups have a significant impact on chromophores. Firstly, there is the impact on the T_g_ of the chromophore itself: the introduction of rigid groups will result in a larger T_g_, such as the anthracene-based chromophore compared to furan-based chromophore. Secondly, the glass transition temperature of the cross-linked material will increase by 50–100 °C compared to the pre-cross-linked material, but there is a significant difference in the improvement of T_g_ due to different reactions. But what needs to be pointed out here, is that the number of cross-linking groups also directly determines the degree of cross-linking. We should consider the number of cross-linking groups when designing cross-linked materials.

The third aspect is the impact on the poling process and electro-optic coefficient. The poling process of cross-linked materials first completes the poling orientation near the glass transition temperature of the material, and then increases the temperature to complete the cross-linking and fix the already oriented molecules. So the cross-linking starting temperature (T_s_) should be near but higher than T_g_, and then the cross-linking completion temperature (T_f_) should not exceed Tg too much; otherwise, excessively high temperatures may cause the thermal movement of molecules to be too intense or destroy the chromophore to some extent. For these four reactions, the cross-linking temperature (135 °C) of the AM-DA reaction was lower and the cross-linking time was shorter (30 min), which is beneficial for accelerating the poling process and improving the poling efficiency.

The biggest obstacle to the commercialization of organic electro-optical materials is their weak stability. The T_g_ is the temperature at which molecular order and electro-optic activity is lost in organic NLO materials. Telcordia standards require operation at 85 °C for 2000 h. So, T_g_ must be well above this threshold to meet this goal. For integration with more complex devices, processing steps after EO poling may require high temperatures. So, thermally stable EO activity at 200 °C would be hugely important. At present, the maximum T_g_ of binary cross-linked electro-optic materials is only 185 °C, which has not yet reached the ideal standard of over 200 °C. Perhaps the T_g_ of the chromophore can be improved by introducing rigid cross-linking groups, such as the bis- anthracene structure. In addition to stability, a simpler cross-linking formula is also very important, as the design of binary cross-linking chromophores involves issues of ratio and chromophore matching. Perhaps integrating cross-linkable groups into a single chromophore to form a single-component cross-linkable electro-optic material system is more conducive to industrial production.

**Figure 10:**
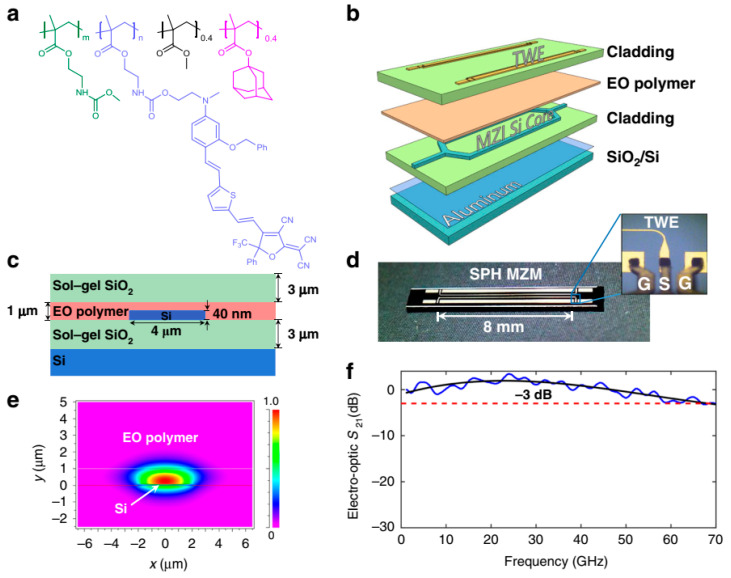
(**a**) Molecular structure of the synthesized side-chain EO polymer. (**b**) Schematic diagram of the fabricated SPH modulator by layers. (**c**) Cross-sectional view of the phase shifter section of the SPH modulator. (**d**) Top view photograph of a fabricated SPH modulator chip. (**e**) Numerically simulated optical field distribution in the cross-section of the modulator. (**f**) Measured EO bandwidth of the SPH modulator.

Another type of organic electro-optic material with high T_g_ is the side chain polymer [16,61,99]. Side chain-type electro-optic polymers connect organic chromophore molecules as side chains to the main chain of the polymer in the form of covalent bonds. The glass transition temperature T_g_ of these systems is usually higher than that of host–guest doped systems with the same chromophore concentration. The polarization orientation relaxation is slower. This type of electro-optic polymer system has a high concentration of chromophores and is not prone to crystallization and phase separation. As for the synthesis methods of such polymers, they can also be divided into two types: direct polymerization and post functionalization. The former involves preparing nonlinear chromophore monomers, and then directly copolymerizing chromophore molecules with other monomers through polymerization reactions [100]. The latter involves preparing polymers and chromophore molecules containing reaction sites separately, and then connecting the chromophore molecules to the polymer through chemical reactions [101]. The advantage of direct polymerization is that the amount of chromophores can be controlled. The advantage of post functionalization is that it can hook different chromophores. The attachment of chromophores can be perpendicular to or parallel to the polymer based on the reaction site. The Yokoyama group developed side chain EO polymers with an ultra-high T_g_ of up to 172 °C, high electro-optic (EO) activities (in-device n_3_r_33_ = 1021 pm V^−1^), and low propagation loss (α, 0.22 dB mm^−1^) as shown in Figure 10, [29]. This polymer was using to fabricate silicon–polymer hybrid modulators which support ultra-fast single-lane data rates up to 200 GB/s. The silicon–organic hybrid (SOH) modulators demonstrated excellent, strong stability and can operate stably for over 2000 h at 110 °C. Although side chain polymers with excellent performance have been designed and synthesized, as the poling temperature approaches the decomposition temperature of the chromophore, this will to some extent cause the decomposition of chromophores, and the problem with the yields of side chain chromophores covalently attached to high T_g_ polymers still needs to be solved. In addition, there are fewer types of chromophores in side chain polymers, and introducing chromophores with higher first-order hyperpolarizability into side chain polymers may improve the electro-optic coefficient of the side chain polymers.

## 5. Application of Electro-Optic Materials: High-Performance EO Modulation

Many organic electro-optic materials have been successfully applied to various advanced optoelectronic devices, including cross-linked materials HLD1/HLD2 [6,87], side chain polymer electro-optic materials [29], the pure chromophore materials JRD1 [30] and DLD164 [102], and the binary material HD-BB-OH/YLD124 [103]. Silicon−organic hybrid (SOH) and plasmonic−organic hybrid (POH) technologies have been used in a Mach−Zehnder modulator (MZM) to improve the electro-optic (EO) performance relevant to both digital and analog signal processing. The π-voltage−length (U_π_L) is an important device figure of merit for electro-optic modulators. The U_π_L can be used to measure the response speed and voltage tolerance of semiconductor devices. A smaller half wave voltage length product means that the voltage can be transmitted better, while a larger half wave voltage length product means that the device has a lower tolerance for voltage. Reductions in U_π_L also mean more compact devices. The U_π_L of the lithium niobate modulators was usually ≥10 Vcm. As shown in Table 1, the U_π_L of the SOH modulators using the side chain polymers, JRD1 and DLD164, was smaller than 1.44 V cm, while the U_π_L of the POH modulators using HLD1/HLD2 and HD-BB-OH/YLD124 was further optimized to <120 Vµm. A smaller U_π_L means a smaller device size, making it easier to integrate at the chip level, which was a huge advantage of organic materials. The refractive index n is also an important parameter that affects the performance of the device. From Table 1, we can see that the n of organic electro-optic materials ranges from 1.66 to 1.88, with the refractive index of pure chromophores slightly higher than that of polymer electro-optic materials. The increase in the refractive index of materials is beneficial for the improvement of device performance, as n has a strong influence on the in-device figure of merit (FOM) for quantifying the efficiency of optical modulation (n^3^r_33_). The larger the n^3^r_33_ value, the smaller the U_π_L value.

In addition to the parameters that affect the transmission rate of the device, stability is also an important factor. The actual usage scenarios of devices may have high temperature and humidity environments, so the communication industry requires communication devices to operate stably at 85 °C for at least 2000 h. The main factor affecting the stability of modulators based on organic electro-optic materials is the glass transition temperature of the organic electro-optic materials. Once the usage temperature exceeds the glass transition temperature of the material, the chromophore molecules that have already polarized under the action of the electric field will become non-centrosymmetric and lose their electro-optical activity. So the temperature at which the device can operate stably is at least 30 °C lower than the T_g_ of the chromophore. For example, the glass transition temperature of the cross-linked material HLD1/HLD2 was 174 °C, and the device can operate stably at a maximum temperature of 140 °C. The glass transition temperature of pure chromophores such as JRD1 and DLD164 is lower than 85 °C, so the device is only suitable for operation at room temperature. Of course, there are other factors that affect the stability of the device, such as high humidity environments and optical stability. At present, there is relatively little research on these factors, which will be a future research direction.

## 6. Conclusions

Organic electro-optic materials have the advantage of a high electro-optic coefficient (~1000 pm/V) and could allow the utilization of photonic devices for the chip-scale integration of electronics and photonics, as compared to inorganic electro-optic materials. It is precisely because of these advantages that many advanced optoelectronic devices based on organic electro-optical materials have been manufactured, and some materials have commercial application prospects. But the true commercialization of organic materials still has a long way to go. Here, two issues need to be addressed: Firstly, there is a need to design and synthesize chromophore materials with large electro-optic coefficients; although chromophores with an electro-optic coefficient exceeding 1000 pm/V have been developed, the electro-optic coefficient of most chromophores is still below 300 pm/V. The solution lies in developing new chromophores and enhancing poling processes. Secondly, commercial communication materials must meet the Telcordia GR-468-CORE standards; the biggest disadvantage of organic electro-optical materials compared to inorganic materials is their insufficient stability, especially for long-term poling orientation stability. The T_g_ is the temperature at which molecular order and electro-optic activity is lost in organic NLO materials. Telcordia standards require operation at 85 °C for 2000 h. So, T_g_ must be well above this threshold to meet this goal. For integration with more complex devices, processing steps after EO poling may require high temperatures. More methods can be explored to improve T_g_, such as developing new cross-linking methods for electro-optic materials, improving the rigidity of small molecules in organic electro-optic materials, and so on. At present, the maximum T_g_ of binary cross-linked electro-optic materials is only 185 °C, which has not yet reached the ideal standard of over 200 °C. Perhaps the T_g_ of the chromophore can be improved by introducing rigid cross-linking groups, such as the bis-anthracene structure. In addition to stability, a simpler cross-linking formula is also very important, as the design of binary cross-linking chromophores involves issues of ratio and chromophore matching. Perhaps integrating cross-linkable groups into a single chromophore to form a single-component cross-linkable electro-optic material system is more conducive to industrial production. For side chain-type electro-optic polymer materials, how to increase the glass transition temperature while solving the problem of poor solubility, and how to introduce chromophores with higher first-order hyperpolarizability into the polymer system to obtain larger electro-optic coefficients are also problems that need to be solved.

## Figures and Tables

**Table 1:** Some parameters of EO modulators based on organic electro-optic materials.

EO Material	T_o_^a^ (°C)	Device Type	T_g_ (°C)	U_π_L	n ^e^	n^3^ r_33_ (pm/V)	r_33_ in Device (pm/V)
HLD1/HLD2	140	POH ^c^	174	120 Vµm	1.83	3100	100
side chain polymers	140	SOH ^d^	172	1.44 V·cm	1.66	1021	223
JRD1	RT ^b^	SOH ^d^	82	0.32 Vmm	1.81	2313	390
DLD164	RT ^b^	SOH ^d^	66	0.5 Vmm	1.88	1103	180
HD-BB-OH/YLD124	<80	POH ^c^	110	10 Vμm	1.73	1220	220

^a^ Stable operating temperature; ^b^ room temperature; ^c^ silicon-plasmonic modulator; ^d^ silicon–organic hybrid modulator; and ^e^ refractive index n @ 1.55 μm.

## Data Availability

No new data were created or analyzed in this study.
